# Individual MHCI-Restricted T-Cell Receptors are Characterized by a Unique Peptide Recognition Signature

**DOI:** 10.3389/fimmu.2013.00199

**Published:** 2013-07-23

**Authors:** Linda Wooldridge

**Affiliations:** ^1^Institute of Infection and Immunity, Cardiff University School of Medicine, Heath Park, Cardiff, UK

**Keywords:** MHCI-peptide length, T-cell crossreactivity, vaccination, autoimmunity, alloreactivity

## Abstract

Effective immunity requires that a limited TCR repertoire is able to recognize a vast number of foreign peptide-MHCI (peptide-major histocompatibility complex class I) molecules. This challenge is overcome by the ability of individual TCRs to recognize large numbers of peptides. Recently, it was demonstrated that MHCI-restricted TCRs can recognize up to 10^6^ peptides of a defined length. Astonishingly, this remarkable level of promiscuity does not extend to peptides of different lengths, a fundamental observation that has broad implications for CD8^+^ T-cell immunity. In particular, the findings suggest that effective immunity can only be achieved by mobilization of “length-matched” CD8^+^ T-cell clonotypes. Overall, recent findings suggest that every TCR is specific for a unique set of peptides, which can be described as a unique “peptide recognition signature” (PRS) and consists of three components: (1) peptide length preference, (2) number of peptides recognized; and, (3) sequence identity (e.g., self versus pathogen derived). In future, the ability to de-convolute peptide recognition signatures across the normal and pathogenic repertoire will be essential for understanding the system requirements for effective CD8^+^ T-cell immunity and elucidating mechanisms which underlie CD8^+^ T-cell mediated disease.

## Introduction

CD8^+^ T cells are important for the control of viral infection and the natural eradication of cancer. CD8^+^ T cells recognize short peptides presented at the target cell surface bound to major histocompatibility complex class I (MHCI) molecules (1). Recognition of peptide-MHCI (pMHCI) is mediated by the alpha beta T-cell receptor (TCR). Initially, it was thought that MHCI molecules predominantly presented peptides of between 8 and 10 amino acids in length. However, subsequent studies demonstrated that peptides of between 11 and 14 amino acids in length are also presented and can be highly immunogenic (2). In fact, many longer peptides can be immunodominant over completely overlapping shorter peptides that also bind to the same MHCI allele (3–6). Thus, it is now well established that MHCI can present peptides of between 8 and 14 amino acids in length. A vast array of peptides within this length range can be generated from permutations of the twenty proteogenic l-amino acids (1.7 × 10^18^). Of course, not all of these peptide sequences occur in the current database of natural proteins. However, it is vital that the TCR repertoire is poised to recognize vast numbers of peptide sequences, even if not identified to date, in order to adapt and deal with changes in pathogen diversity.

The MHCI-presented peptide repertoire is constrained by factors such as the specificity of antigen processing pathway components (proteasome, ERAAP/ERAP1, tapasin, and TAP) and the HLA type of the individual (7, 8). As a result, conservative estimates suggest that only ∼1% of the peptide universe can be processed and presented at the cell surface in the context of MHCI (9). However, this still leaves a staggering 1.7 × 10^16^ peptides of different lengths to which a relatively small number of TCRs (∼25 million) (10) must be able to respond appropriately. Therefore, in order to understand the requirements for effective CD8^+^ T-cell immunity, it is important to understand how a limited TCR repertoire responds and adapts to the vast number of possible different epitopes across a length spectrum.

## Individual TCRs Can Recognize Large Numbers of Peptides of a Defined Length

It has been appreciated for many years that individual TCRs can recognize multiple ligands (11–18). Initial studies of TCR degeneracy were conducted using small numbers of peptide analogs (typically less than 200). The development of combinatorial peptide library (CPL) screening which was pioneered by Houghten et al. (19, 20) allowed for a more comprehensive analysis of TCR degeneracy and the ability to define much larger numbers of candidate peptide analogs (21). A more recent study examined ∼4,000 peptides, although it must be appreciated that this number still only represents an extremely small portion of the entire peptide universe (18).

Estimates of TCR degeneracy were initially performed using data from peptide library screening. Elegant studies which were conducted by two independent groups estimated that individual MHCII-restricted TCRs can recognize ∼10^6^ peptides (22, 23). A similar analysis performed by Wilson and colleagues suggested that MHCI-restricted TCRs exhibit lower levels of degeneracy (24). However, approaches performed using small peptide sets or library screening alone preclude a comprehensive analysis of the entire peptide universe and as such, may result in an underestimate of TCR degeneracy. In order to address this possibility, a recent study developed an approach to quantify TCR degeneracy that can be used to probe the entire peptide universe of a defined length. This approach involves a combination of sampling from motif-restricted peptide sets and sampling from the entire peptide universe with bias toward those peptides predicted to elicit a response (termed CPL-based importance sampling) (25). The approach was used to construct a degeneracy curve which revealed, in the initial application of this strategy, that an individual MHCI-restricted TCR (1E6) can recognize over one million different decamer peptides in the context of a single MHCI at physiologically relevant concentrations. Furthermore, it was possible to identify peptides that were up to 100-fold more potent than the cognate peptide for this particular TCR. To date, this study represents the most comprehensive quantification of MHCI-restricted TCR degeneracy (25). However, continued development of techniques that allow rapid and accurate quantification of TCR degeneracy are essential if we are to understand the role that this phenomenon plays in common T-cell mediated diseases.

The ability of individual TCRs to recognize large numbers of peptides provides an explanation for how a limited TCR repertoire can provide effective immune coverage against all possible foreign pMHCI that can be encountered. However this analysis was confined to a single autoimmune TCR which may represent the extreme end of the cross-reactivity spectrum. In order to understand the system requirements for effective CD8^+^ T-cell immunity, it will be important to measure how this phenomenon varies across the normal TCR repertoire. It is tempting to speculate that variations in levels of TCR degeneracy may contribute to the pathogenesis of disease. In fact there is emerging evidence to suggest that this is indeed the case. For example, several studies suggest that levels of TCR degeneracy can influence disease outcome during infection with viruses such as HIV-1 that have a high mutation rate (26–29). Overall more information is required about how TCR degeneracy varies across the normal TCR repertoire and the role that variations play in the pathogenesis of disease.

## TCRs Exhibit an Explicit Preference for MHCI-Bound Peptide Length

It is clear that TCRs can recognize a large number of peptides of a defined length and it would seem logical that this flexibility could extend to the recognition of peptides of different lengths, as this would maximize the ability of the TCR repertoire to provide broad antigenic coverage. In order to examine this possibility, a recent study utilized a series of combinatorial peptide libraries, ranging from 8 to 13 amino acids in length, to perform a comprehensive analysis of peptide length preference across a panel of CD8^+^ T-cell clones raised against a range of pMHCI antigens spanning different peptide lengths derived from both foreign and self antigens (30). An unexpected feature of the TCR/pMHCI interaction was discovered by showing that any given TCR exhibits an explicit preference for a single MHCI-bound peptide length (30). This MHCI-bound peptide length preference was identical to the length of the original peptide which had been used to derive each clone. Furthermore, this unexpected finding applied to all TCRs examined regardless of antigenic specificity and MHCI restriction. Importantly, peptide length preference was shown to be an intrinsic feature of the TCR and not determined by differential MHCI-peptide binding. A very small number of agonists of non-preferred length were identified, but the incidence of this phenomenon was extremely rare and recognition was generally sub-optimal when compared to peptides of the preferred length. Interestingly, however, agonists of non-preferred length identified were entirely distinct in sequence, with a different amino acid residue at every position. As described above, CPL-based importance sampling revealed that the 1E6 TCR can recognize over one million different 10mer peptides at a functional sensitivity equivalent to or greater than index (25). In contrast, the 1E6 TCR was not capable of recognizing any 8, 9, 11, 12, or 13mer peptides within this functional sensitivity range. Thus, individual MHCI-restricted TCRs exhibit an explicit preference for a single MHCI-peptide length (30). The next challenge will be to explain how a repertoire of TCRs that display a stringent preference for peptide length can provide effective immune coverage against all peptide lengths that can be presented by MHCI. To address this question, it will be important to examine how peptide length preference varies across the entire TCR repertoire.

## Implications of MHCI-Bound Peptide Length Preference for CD8^+^ T-Cell Immunity

The TCR/pMHCI interaction is pivotal in all aspects of CD8^+^ T-cell immunity including thymic development, naive T-cell survival, and recognition of foreign pMHCI in the periphery. Peptide length determines the outcome of TCR/pMHCI engagement and, as such, has far-reaching implications (Box 1). Firstly, the findings predict that the thymic epithelial cell (TEC) (31, 32) surface must display peptides of multiple different lengths to ensure that an appropriately diverse CD8^+^ T-cell repertoire undergoes positive and negative selection before being released into the periphery. Once in the periphery, continual TCR engagement of low affinity self-derived pMHCI molecules is essential for naive T-cell survival (33, 34). Here, peptides of non-preferred length may actually play a very important role because signaling below the threshold for T-cell activation may be sufficient for the delivery of survival signals.

Box 1 **Implications of peptide length preference.**

**A: Implications for CD8^+^ T-cell immunity**

- Thymic development: TECs must display peptides of multiple lengths
- Naive T-cell survival: mediated by recognition of defined length peptides
- Effective CD8^+^ T-cell immunity: requires “length-matched” clonotypes
- Protective vaccination: must elicit “length-matched” clonotypes

**B: Implications for disease pathogenesis**

- Identification of ligands that trigger CD8^+^ T-cell expansions in autoimmunity and hematological disease: length preference must be established
- Rare peptides of non-preferred length peptides with entirely disparate sequence: may represent a novel mechanism underlying molecular mimicry
- Alloreactivity: an important consideration in the identification of alloreactive ligands

Importantly, the findings imply that the CD8^+^ T-cell repertoire is compartmentalized with respect to peptide length preference, such that effective immunity can only be achieved by mobilization of the appropriate “length-matched” antigen-specific CD8^+^ T-cell clonotypes (30). To understand the system requirements for effective CD8^+^ T-cell immunity, it will therefore be necessary to examine how peptide length preference varies across the normal TCR repertoire and how different “peptide length footprints” overlap to provide effective coverage across all peptide lengths. The length preference phenomenon also informs our understanding of the requirements for effective peptide vaccination. For example, it was recently observed that the heteroclitic 10mer epitope ELAGIGILTV derived from MART-1/Melan-A, which is commonly used in melanoma clinical trials, primes a population of CD8^+^ T cells that is very poor at cross-recognizing the dominant naturally presented 9mer epitope AAGIGILTV (35, 36). These findings may explain, at least in part, the poor objective response rates observed in these trials (37). Accordingly, such findings suggest that peptide length is likely to be an important consideration in the provision of effective CD8^+^ T-cell immunity and the response to peptide vaccination.

## Implications of MHCI-Bound Peptide Length Preference for Disease Pathogenesis

As well as playing a critical role in CD8^+^ T-cell immunity, the TCR/pMHCI interaction can result in inappropriate CD8^+^ T-cell activity in situations such as autoimmunity, hematological disorders, and transplant rejection (alloreactivity). Factors that influence the outcome of TCR/pMHCI engagement, such as peptide length, therefore impact on our understanding of disease pathogenesis. Transient, asymptomatic CD8^+^ T-cell expansions are relatively common and often associated with viral infections. However, in certain disease states, monoclonal/oligoclonal CD8^+^ T-cell expansions with a differentiated phenotype persist, suggesting an exaggerated response to an immunodominant antigen. Monoclonal CD8^+^ T-cell expansions are a characteristic feature of T-LGL leukemia (38), but can also be triggered by drugs [e.g., protein tyrosine kinase inhibitors (39)]. Oligoclonal expansions have been observed in various autoimmune diseases [e.g., multiple sclerosis (40, 41), rheumatoid arthritis (42), and aplastic anemia (43)]. To date, there has been no attempt to identify the origin of the ligands that drive these potentially pathogenic CD8^+^ T-cell populations. This is a priority for the future because it will yield information about the pathogenesis of these important diseases. In order to achieve this goal, the dominant pathogenic T-cell clone must be isolated and the peptide length preference defined. This will narrow the search and allow the selection of length-appropriate peptide libraries to enable ligand hunting.

Alloreactivity represents a major barrier to transplantation and, as such, there is a pressing need to understand the molecular and structural basis of this phenomenon (44, 45). This is especially important considering that substantial efforts are being made to increase the number of available donors despite the fact that, in the last decade, little progress has been made in understanding how to reduce the risks and severity of transplant rejection. It is well established that alloreactive T-cells are highly prevalent within the T-cell repertoire. However, the mechanisms that underlie this phenomenon remain unclear. Originally, it was thought that alloreactivity was mediated by TCR recognition of foreign MHC (“MHC-centric”). More recently, however, it has become increasingly apparent that the alloreactive TCR/pMHCI interaction can be highly peptide-dependent (“peptide-centric”) (46–48). In fact, it appears that peptide specificity is profound when self-MHCI and foreign-MHCI are closely related (49). Further studies are required to determine whether alloreactive TCR/pMHCI interactions exhibit the levels of promiscuity that typify autologous TCR/pMHCI interactions. It will also be important to examine the possibility that alloreactive TCR/pMHCI interactions may exhibit a peptide length preference. This will facilitate the identification of alloreactive ligands and allow characterization of the peptide repertoire recognized in the context of non-self MHCI (30).

## Individual MHCI-Restricted TCRs are Characterized by a Unique Peptide Recognition Signature

**Figure 1:**
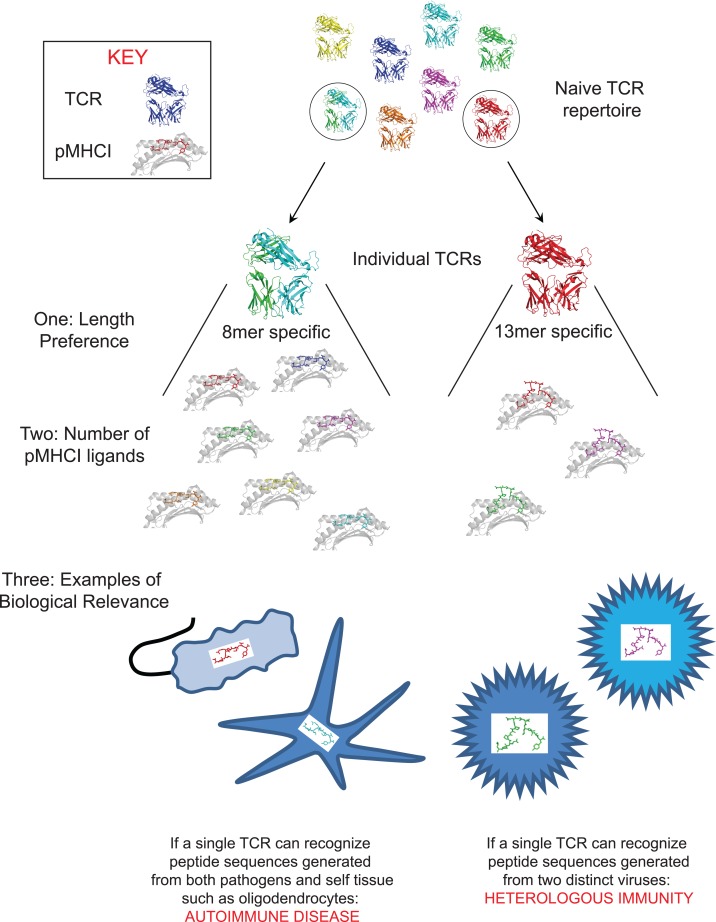
**The peptide recognition signature of every TCR consists of three components**. The figure depicts the three components of the peptide recognition signature. *One: peptide length preference:* this finding implies that effective CD8^+^ T-cell immunity can only be achieved if “length-matched” antigen-specific CD8^+^ T-cell clonotypes are mobilized during an immune response, *two: number of peptides recognized at the preferred peptide length:* which can be very large (up to ∼10^6^). The ability of individual TCRs to recognize multiple pMHCI is essential for the provision of effective immune coverage against the multitude of foreign pMHCI that can be encountered, and; *three: sequence of the recognized peptides (which dictates the biological relevance of the peptide recognition signature):* the ability to be able to recognize both pathogen and self-derived pMHCI could be the basis for “molecular mimicry,” which is the widely hypothesized cause of autoimmune disease. Evidence for this phenomenon exists in animal models of autoimmunity but convincing data in human disease is still lacking. By way of an example, the figure depicts the ability of the same TCR to recognize peptide sequences generated from both environmental pathogens and oligodendrocytes. Alternatively, if a TCR can respond to naturally processed and presented peptide sequences from more than one pathogen then this will allow a single TCR to provide immunity to more than one infection. It has been suggested that this phenomenon (often referred to as “heterologous immunity”) is extremely important for the provision of effective immunity. By way of an example, the figure depicts the ability of the same TCR to recognize naturally processed and presented peptide sequences from two different viruses.

Taken together, recent observations suggest that every TCR is specific for a unique set of peptides, which can be described as a unique “peptide recognition signature” (PRS) (Figure 1). The PRS of individual TCRs comprises three different components: (i) an explicit preference for a single MHCI-peptide length; (ii) the number of peptide sequences recognized at the preferred length, which can be very large (up to ∼10^6^ different peptides); and, (iii) the sequence identity of the recognized peptides, many of which will be biologically relevant. The sequence identity of the peptides recognized by an individual TCR is the most important component of the PRS (Figure 1). Many of the peptide sequences recognized by individual TCRs can appear in a large number of self proteins or pathogen derived proteins. If these sequences can be naturally processed and presented at the cell surface then the TCR will have the opportunity to mount a response to them *in vivo*. If a TCR can respond to naturally processed sequences from more than one pathogen then this will allow a single TCR to provide immunity to more than one infection. This phenomenon [often referred to as “heterologous immunity” (50, 51)] is extremely important for the provision of effective immunity and an example of the immense benefits that can be gained from TCR degeneracy. However, the ability to be able to recognize both self and pathogen derived pMHCI has the potential to be highly pathogenic and provides a mechanistic basis for “molecular mimicry,” which is the widely hypothesized cause of autoimmune disease (52). Although good examples of molecular mimicry exist in mouse models of disease there is still a lack of concrete evidence for this in human autoimmune disease (52). It is very important that this question is answered in the context of human disease. The ability to de-convolute the PRS of TCRs involved in autoimmune disease will be of immense interest in the future.

## Approaches that Can be Used to De-Convolute the PRS of Individual TCRs

CD8^+^ T cells play a major role in the pathogenesis of autoimmunity, hematological disease, and transplant rejection, as described above. In the future, the ability to de-convolute the PRS of pathogenically relevant TCRs will allow us to dissect the underlying mechanisms of common CD8^+^ T-cell mediated disease. This information can then be used to inform the rational design of novel therapeutic strategies. As such there is a pressing need to design approaches that can be used to achieve this goal. There have been some elegant attempts to develop an approach that allows the identification of ligands recognized by CD8^+^ T-cell populations of potential pathogenic significance (15, 53, 54). However, an appreciation that every TCR is characterized by a unique PRS will facilitate this analysis. Firstly, it will be necessary to define the peptide length preference of the individual TCR of interest so that appropriate ligand hunting tools can be selected, such as length-matched peptide libraries. Secondly, the number of peptides that can be recognized by the individual TCR may influence the approach that is being used. For example, highly cross-reactive TCRs will be more challenging than those that exhibit a more focused phenotype. And finally, tools to identify the origin of the peptide sequences (i.e., pathogen versus self protein) will be essential. Emphasis should be placed on developing an approach that is rapid and can be scaled up to allow the analysis of large numbers of TCRs in a short space of time. If this can be achieved, then the ability to de-convolute PRSs across the normal and diseased repertoire is expected to yield large but very important databases of information for the future.

## Concluding Remarks

In summary, based on recently generated data, I suggest that every TCR in the naive repertoire is characterized by a unique PRS; all of which overlap to provide effective immune coverage against all possible foreign-MHCI-bound peptides that could be encountered. A comprehensive study to examine how the PRS of individual TCRs varies across the normal TCR repertoire will allow us to determine how a relatively limited number of TCRs can recognize the multitude of 8–14 amino acid length peptides that can be encountered in complex with MHCI, and thereby define the system requirements for effective CD8^+^ T-cell immunity. In addition, approaches that can be used to de-convolute the PRSs of individual pathogenically relevant TCRs are essential if we are to gain an understanding of the mechanisms that underlie common CD8^+^ T-cell mediated diseases.

## Conflict of Interest Statement

The authors declare that the research was conducted in the absence of any commercial or financial relationships that could be construed as a potential conflict of interest.
